# A program evaluation of the Innovative Teen Pregnancy Prevention Programs (iTP_3_) Project

**DOI:** 10.1186/s13690-021-00723-z

**Published:** 2022-01-10

**Authors:** Kristen M. Garcia, Christi H. Esquivel, Whitney R. Garney, Kelly L. Wilson, Jennifer Farmer

**Affiliations:** Department of Health & Kinesiology, Texas A&M University, College Station, TX USA

**Keywords:** Innovation, Program design, Program planning, Adolescent health, Evaluation

## Abstract

**Background:**

Teen pregnancy prevention in the United States has traditionally focused on the development, testing, and subsequent implementation of a set of evidence-based programs (EBPs), recommended nationally. However, these existing EBPs often do not prioritize the most at-risk or vulnerable populations.

**Methods:**

The Innovative Teen Pregnancy Prevention Programs (iTP_3_) project was funded to facilitate the development of new, innovative programs to reach disparate populations. Through a mixed methods design, iTP_3_ evaluated the process and resulting innovative programs from five iterative cohorts of funded organizations, referred to as Innovators. iTP_3_ utilized both a traditional funding model with more traditional methods of capacity building assistance, but transitioned over time to a design-focused funding model in which organizations and individuals developed innovative programs through an intensive human centered design process.

**Results:**

Evaluation results showed that the resulting portfolio of programs had differences in the types of programs resulting from the differing funding models. Notable differences among programs from the two funding models include program length, along with personnel, time, and resources needed to develop and manage.

**Conclusion:**

Both traditional and design funding models led to innovative programs, with notable differences in the development process and resulting programs.

## Background

Federal support for teen pregnancy prevention and adolescent health programs has made significant progress in the last two decades. In 2007, the first list of evidence-based programs (EBPs) for teen pregnancy prevention (TPP) was released, which identified 15 programs shown to delay sexual debut, increase contraception use, and decrease teen births [1]. Three years later, in 2010, governmental entities issued funding opportunities to support implementation of existing EBPs, rigorous evaluation of newly developed programs, and the development of new programs [2, 3]. These priorities continued with two subsequent funding opportunities in 2015 and 2020.

As of 2017, there are over 40 EBPs for TPP, which have contributed to the decrease in teen birth rates in the United States [4, 5]. However, as teen births have hit an all-time low [5], disparities among some subpopulations and geographic regions are highlighted [6–9].

Current TPP EBPs, which focus on reducing sexual risk behaviors including sexual initiation, number of sexual partners, frequency of sexual activity, contraception use, pregnancy, and sexually transmitted infections, prioritize limited underserved subpopulations [4]. Many programs fail to address dynamic contextual and environmental factors that go beyond the individually focused sexual risk behaviors previously listed. Other potential interventions that go beyond an individual-level focus include, but are not limited to: relationships with partners, social norms, and availability and access to contraception [10–12]. This presents a need for new programs developed for those most at risk. In an effort to effect the most disparate populations, federal agencies including the Office of Population Affairs (previously the Office of Adolescent Health) and the Family and Youth Services Bureau have issued funding for innovative approaches to TPP and adolescent health [13, 14].

### iTP_3_ project description

The Innovative Teen Pregnancy Prevention Programs (iTP_3_) project, led by Texas A&M University, was funded in 2015 by the U.S. Department of Health and Human Services’ Office of Population Affairs (OPA) (then the Office of Adolescent Health), to support and enable innovation in TPP through a 5 year funding period [2]. Innovation, in this project, prioritized disparities in TPP and focused on underserved communities, racial and ethnic minority groups, and other at-risk populations.

The iTP_3_ project acted as an intermediary by first identifying organizations across the United States who sought to develop innovative TPP programs, then provided funding and capacity-building assistance (CBA) to support their program development work. iTP_3_ defined programs as, “A systematic strategy (activity(s), policies, procedures, interventions, etc.) that can be, or are, replicated or repeated in one or more settings.” Twenty-seven unique organizations, across 5 cohorts, were funded through the iTP_3_ project from 2015 to 2020. Annual funding amounts ranged from $8500 to $100,000. Funding amounts varied because each year iTP_3_ altered its funding structure based on lessons learned. Initially, iTP_3_ utilized a traditional grantee model (cohorts 1 and 2), which identified recipients and provided funding for independent activities. Individualized CBA was provided by the iTP3 project team and external experts on a case-by-case basis, based on Innovator requests and needs identified by iTP_3_ personnel. Potential CBA focused on target populations, program design, implementation, evaluation, and other topics.

Cohorts 3–5 utilized a design-focused funding model, which sought to build organizational capacity through developing skills in human centered design (HCD). HCD is a process that includes building empathy for the target population, generating ideas, developing prototypes, testing, and iterating products or programs with a goal of developing solutions to be implemented [15]. Cohorts 3–5, were funded to participate in intensive CBA to develop program idea(s), then received smaller funding amounts to operationalize and pilot test their programs. Additional CBA for cohorts 3–5 focused on systems thinking which included training on systems concepts and ecological perspectives, developing systems maps, and assistance in incorporating systems thinking concepts in the program design. All iTP_3_ funded organizations are referred to as Innovators throughout this manuscript. See Table 1 for a description and timeline of iTP_3_ cohorts.

**Table 1:**
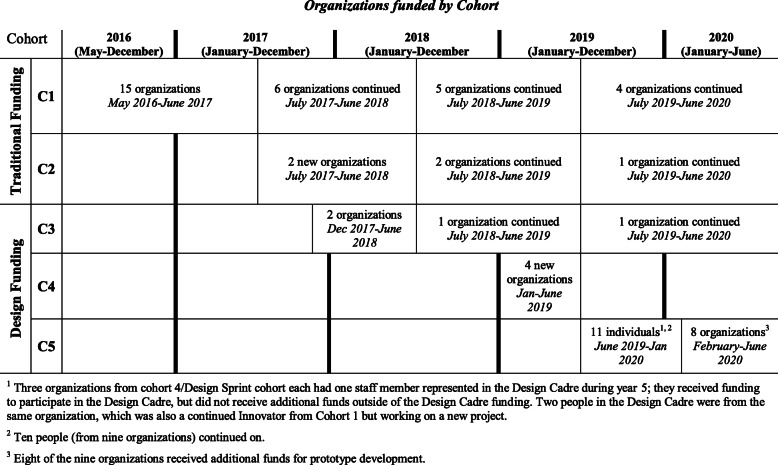
Overview of Organizations funded by Cohort

The approach to support Innovators evolved based on continued quality improvement efforts. Common methods of CBA delivery included webinars, conference calls, online modules and resources, learning collaboratives, peer sharing calls, site visits, virtual meetings, and in-person trainings. CBA providers were determined based on Innovators’ needs and included members of the iTP_3_ team as well as external subject matter experts. Group- and individual-level CBA was available each year but differed among cohorts. Innovators in the design-focused cohorts received much more intensive CBA in comparison to the more traditional cohorts as they were required to participate in trainings whereas the traditional cohorts only received CBA on an as needed basis. A detailed description of the iTP_3_ model of innovation will be available in other literature.

To understand the implementation and outcomes of the iTP_3_ project, a program evaluation was conducted. While the evaluation explored many aspects of the iTP_3_ project, the primary evaluation question aligned with the funding objective, which was: *To what extent did iTP3 result in the development of a portfolio of innovative teen pregnancy prevention programs?* The purpose of this paper is to report on that evaluation question and provide insight on innovative program development in public health.

## Methods

To capture the most relevant information, the evaluation was designed to be formative and process-based, utilizing a mixed methods approach (Creswell & Plano Clark, 2018). Due to the nature of this project, 28 organizations (*N* = 28) participated in various data collection activities, rather than having individual participants. Organizational representatives accounted for each of the Innovator entities funded through iTP_3_. These representatives consented to participate in evaluation activities on behalf of their organization, and all data collection tools were approved by the Texas A&M University Institutional Review Board prior to implementation. The evaluation was conducted by an evaluation team, internal to the iTP_3_ project.

### Data collection tools

Given the length and scope of the iTP_3_ project, multiple data sources were developed to capture information regarding the program objectives. Data collection tools were altered to accommodate changes in the funding models and CBA. See Table 2 for a detailed description of each data collection tool and the participating cohorts.

**Table 2:** iTP3 Data Tools and Description

Source	Purpose	Cohort(s)	Timeline
**Interviews (Program Development)** *Qualitative Source*	Assess program planning, development, and implementation process; innovation; and successes and challenges experienced by Innovators.	1, 2, 3	End of each program years 2–4
**Interviews (Design Sprints)** *Qualitative Source*	Assess use of HCD and systems thinking in the program development process and applicability of design skills outside developing their program.	4	End of program year 4
**Interviews (Design Cadre)** *Qualitative Source*	Assess participants’ perceptions of the design process, reflections of the training they received, self-efficacy for leading a Design Sprint, their resulting program, innovation, and other relevant domains.	5	End of program year 5
**Innovator Database** *Quantitative Source*	Track program development process (including activities completed); stakeholder participation and engagement; program materials created; and program characteristics.	1, 2, 3	Monthly, years 2–3
**Design Bootcamp After-Action Report** *Qualitative Source*	To record the HCD Bootcamp process including what worked well and what would be more efficient, outcomes of the Bootcamp, key discussions and feedback from participants, and next steps.	3	Following each Design Bootcamp, year 3
**Design Sprint After-Action Reports** *Qualitative Source*	To document design activities (including descriptions and outcomes), key points of discussion, pivot points in the design process, and next steps.	4	Following each Design Sprint Session, year 4

#### Qualitative data collection and analysis

Several types of qualitative data sources, both formal and informal were collected and analyzed. First, qualitative telephone interviews were conducted with all cohorts of Innovators. Interview scripts differed slightly among cohorts due to the varying cohort structures and CBA provided. Additionally, after-action reports were compiled following design sessions to document the overall process with each design team and keep track of pivots in the design process and why decisions were made. The after-action reports were analyzed as archival documents.

A thematic analysis using open-coding to identify emergent themes was used to assess qualitative data sources [16]. Four members of the evaluation team participated in each round of analysis. All members were trained in qualitative data analysis and followed an analysis protocol developed by the evaluators. Evaluators reviewed each data source independently, identified units of data, and organized the datum by common theme [17]. Following independent analyses, evaluators met to review units of coded data and identify themes. This coding process ensured consistency among study findings [16]. Upon disagreement in coding, evaluators sought opinions of another team member.

After completing thematic analyses, findings for each data source were presented to the lead evaluator to review for completeness [18]. Four evaluators, including the lead, then triangulated the data from each data source to look for patterns and common findings. Key findings from all sources are provided in the results section.

#### Quantitative data collection and analysis

Information was collected from Innovators in Cohorts 1–3 on a monthly basis using a Microsoft Access database. The database collected information regarding program characteristics, design and implementation activities, and dissemination of information. The evaluators compiled summary reports within the Access software. This information was used to gauge overall progress and status of program development. The Innovator database which was analyzed using central tendency statistics to understand the extent that activities were conducted.

## Results

Throughout iTP_3_, data from each year was used for continuous quality improvement (CQI) and aided in updating the program model. Results are formatted to show the evolution of the project based on CQI processes.

### Traditionally-funded innovators (cohorts 1–3)

Seventeen Innovators were funded across 2016–2019, using a traditional funding model. Each year, Innovators submitted an application for continued funding based on their progress and the viability of their projects. Four Innovators were engaged for the entire 4-year time period, 2 for 3 years, 2 for 2 years, and 9 for a 1-year period.

Innovator programs represented a variety of priority populations including ethnic and racial minority youth; older teens; pregnant and parenting teens; lesbian, gay, bisexual, transgender, queer and questioning (LGBTQ+) youth; youth in foster care; youth with intellectual and/or developmental disabilities; depressed teens; homeless and runaway youth; and pre-service teachers. Programs also targeted ecological levels of intervention including interpersonal relationships, organizational interventions, community-based-intervention, and individual behaviors. As was expected in the initial funding of innovative interventions, not all programs were fully developed for testing. Rather some only made it through the initial stages of exploration or program development. See Table 3 for a full description of Cohort 1 Innovators.

**Table 3:** Overview of Traditionally-Funded Innovators

Innovator	Priority population	Setting	Years Funded				Innovation/progress notes
			Y2	Y3	Y4	Y5	
1	Older depressed teens	Clinic-based	X	X	X	X	Developed and implemented program for depressed young women utilizing a texting modality; Received funding for rigorous testing
2	Ethnic and racial minority, family units with an adolescent, older teens	Clinic-based	X	X	X	X	Built out a clinic-based intervention focusing on an initial reproductive health care visit, a contraceptive coaching program, and parent and peer advisor program.
3	Ethnic minority, family units with an adolescent, rural populations	Faith based organizations, in school, out of school/community based	X				Cultural adaptation of an EB intervention targeting caregivers and youth; Aims to address generational and cultural barriers by leading parents/trusted adults and youth through talking to each other about sex, relationships and protection, and providing resources and step-by-step supports to encourage and enable conversations.
4	Ethnic and racial minority	Out of school/ community based	X	X			Mentorship program for African American males, in partnership with national African American fraternity.
5	Adolescents who have given birth, LGBTQ, older teens	Institutions of higher education	X				Program designed to reach college freshman with sex education by training and supporting resident assistants (RAs) as sexual health educators.
6	Adolescents who have given birth	In school, out of school/community- based	X				Formative research study to inform the development of an intervention for youth who are pregnant or parenting.
7	Adolescents who have given birth	Out of school/ community-based	X				Information gathering on the feasibility of a peer mentoring program for pregnant and parenting youth.
8	Older teens, youth experiencing Intellectual/ Developmental Disabilities (I/DD)	Unknown- TBD based on assessment feedback	X	X	X		Sustainable, rights-based capacity building program for the provision of comprehensive, consistent, and individualized sexual health education for young people experiencing I/DD; Promote development of skills for young people experiencing I/DD to make informed decisions, increase safety, and seek mutually fulfilling relationships.
9	Youth in foster care	Out of home care settings	X				Background research for a systems-level intervention targeting youth in foster care to improve sexual health information at the individual, interpersonal and policy levels
10	Ethnic and racial minority, older teens, rural populations	In school (high school), outdoors, out of school time and community-based	X				Prepared for pilot testing of a culturally guided TPP program geared toward American Indian youth; utilizes adventure- based experiential learning and service-learning activities; includes a curriculum focused on sexual and reproductive health and healthy relationships.
11	Ethnic and racial minority, LGBTQ, older teens	Out of school/ community-based	X				Development of a program that targets LGB+ youth through education and skill-building to prevent unintended pregnancy; Sessions review sexual orientation, identity and behavior, anatomy, birth control, communication, and influence of environmental factors
12	Older teens	Unknown- TBD based on assessment feedback	X				Gathered input from 18- and 19-year-old cisgender women about perceived and experienced obstacles to accessing SRH information and services; Inform the creation of a product to facilitate access to online or in-person sexual and reproductive healthcare.
13	Youth in foster care	Out of home care settings	X	X	X	X	Capacity building through a multi-level approach to training foster care professionals, creating organizational policies and practices that support SRH education and referrals, and enhancing the physical environment of foster care agencies.
14	Ethnic and racial minority, older teens	Unknown- TBD based on assessment feedback	X				Development of a culturally appropriate multi-component school-based teen pregnancy prevention intervention for African-American older male teens living in economically disadvantaged areas.
15	Adolescents who have given birth, ethnic and racial minority, homeless or runaway, LGBTQ, older teens, victims of intimate violence	Clinic based, outdoors, out of school/ community-based programs, runaway and homeless youth settings	X	X	X	X	Development and testing of a sexual health program targeting homeless and runaway girls. Includes increasing sexual health knowledge, support services to address biopsychosocial and emotional needs, access to medical care, increased connectedness through staff and peer, and tech-based incentives to ensure ability to connect and reach out for services.
16	Pre-service teachers	Institutions of higher education		X	X		Training of pre-service teachers on sex education as part of their teacher training
17	Older teens	Clinic-based, high schools, institutions of higher education		X	X	X	Connect older youth to sexual and reproductive health services as they transition from high school to college or the next step.

Consistent with a traditional funding model, all Innovators began the program with a defined program idea which they planned to implement. The planned programs were at differing stages of development; however due to the nature of the initial application, all Innovators had a defined target population and at least the start of what they perceived to be an innovative idea to build out. Beginning with Cohort 2, began to incorporate some HCD activities, concepts, and training with traditional Innovators. However, given their program development progress, HCD was used more similarly to CQI to iterate portions of the programs rather than develop full ideas.

### Interviews

#### Cohort 1

All 15 funded Cohort 1 Innovators were interviewed regarding their program and their experience with iTP_3_. All Innovators had experience with TPP and most had previous experience with program development. They used this experience to address a gap identified from past programming, apply lessons learned from past programming to their iTP_3_ program, and apply or adopt a TPP program for a new priority population.

Additionally, all Innovators considered themselves to be innovative thinkers. Innovators planned to push innovation further through engaging partners, working with iTP_3_ and CBA providers, and utilizing their organization’s embedded culture of innovation. Long-term, they hoped to have a program ready for a large-scale trial, improve adolescent health, have a new program component, and/or have a program that could be implemented in other communities.

Despite experience with both TPP and program development, Innovators identified seven primary challenges to their iTP_3_ program. These challenges were related to their 1) priority population, 2) ability to develop and disseminate a program, 3) organization, 4) advisory board, 5) partners, 6) ability to replicate the program, and 7) ability to plan for sustainability.

#### Cohort 2

Cohort 2 included eight total Innovators; six returning from year two and two new. All eight innovators completed interviews. Interviews assessed the program planning and implementation process, innovation, and successes and challenges experienced by Innovators. Overall, Innovators felt their core definition of innovation in TPP stayed largely the same but expanded, meaning that they still considered their target population and/or setting innovative, but they began to appreciate more the need to include new strategies, stakeholder input, and often a more holistic approach to solving the problem. Innovators reflected their overall long-term goals remained the same as indicated in their initial applications; however, their process of reaching the end goal changed in some cases. Innovators felt they made progress toward reaching their goals, but more work was needed. This is reflective of this iterative process and push for HCD and systems thinking that was a core aspect of iTP_3_.

There was recognition that innovation is a process rather than an outcome. Innovators recognized that innovation required flexibility and openness to making changes, as well as collaboration with others. In addition to reflecting on their particular programs, Innovators expressed appreciation for the flexibility of the funding model as the ability to make changes and iterate programs is crucial for development of an innovative approach. From the beginning of the grant, OPA allowed iTP_3_ to alter the typical individually focused TPP metrics to focus more on the program development process and encompass non-individually focused interventions. This flexibility from the top allowed project teams a unique opportunity to focus on development and truly meet the needs of their priority population, as opposed to being held to strict, unchangeable metrics and ideas initially proposed. While Innovators were required to develop work plans at the beginning of their project period, it was emphasized that the work plan was an iterative document and should change based on lessons learned, target population input, data, and other relevant factors.

In year four, seven Innovators, all funded the previous year, continued another year of program development. All seven of these Innovators were interviewed, once again. Key findings built upon findings from previous interview iterations. Innovators felt their definition of innovation and how their program was innovative had not changed, but rather solidified throughout the past year. They were still largely working toward the same goal(s) identified in their initial interviews in year two or three (depending on Innovator), but in some cases had expanded their goal to incorporate new elements and reflect lessons learned and/or priority population feedback. Continuing Innovators largely identified HCD and systems thinking as useful and were able to incorporate it broadly into their program development, but several felt the concepts would have been more useful if introduced during their first funding cycle (before the concepts became the focus of iTP_3_ CBA). Overall, these Innovators appreciated the flexibility of this funding model and the opportunity to develop an innovative program through a mechanism that allowed for iteration based on feedback and results. They felt iTP_3_ and innovation funding in general was important to advance the field through reaching underserved populations, fostering cross-sector collaborations, community relevance, and the opportunity to look at problems in new ways and develop creative solutions.

#### Post funding follow-up

In the final project year, six previously funded Innovators who were funded over multiple cohorts were contacted for follow-up interviews. All six who were contacted agreed to be interviewed. It was not feasible to include all Cohort 1 innovators in the follow up interviews as most had not been in contact with iTP_3_ since their funding ended and many had staff turnover of those who were involved in the iTP_3_ program funding. The interviews focused on the status of their program post funding, and overall perceptions of iTP_3_. At the time of the interview all interviewees reported they were able to continue development and expansion of the program. Four reported they completed a pilot of their program and two were ready for rigorous testing. Following iTP_3_ funding, Innovators sought external funding and disseminated their work through peer reviewed publications, dissemination through a website, community presentations, conference presentations, and internal organization conferences.

Innovators were asked specifically how iTP_3_ helped in program development. Innovators reported that administratively, iTP_3_ helped with keeping the program on track. However, beyond administrative functioning, Innovators found that iTP_3_ made a contribution to their program development through sharing HCD and systems thinking technical assistance, funding, guiding the development and iteration processes, encouraging or requiring engagement of stakeholders, and facilitating sharing or collaboration amongst Innovators.

As the years of iTP_3_ progressed, iTP_3_ had a greater emphasis on both HCD and systems thinking as technical assistance for Innovators. Most Innovators indicated they planned to use HCD or systems thinking in the future, while another indicated they hoped to use the techniques but were not as certain. When talking about HCD, most highlighted specific tools and use of the process of design including continuous testing and iteration, and the development of a program that meets the needs of the priority population. For systems thinking, Innovators identified benefits of systems thinking including getting multiple perspectives and stakeholders, looking at the big picture, creating a systems map, and identifying leverage points. Additionally, some Innovators identified specific applications of systems thinking within their work.

### Program model iteration one

Following Cohort 1 and 2, there was an observed need to explore new program development and funding models. As noted through the interviews, Innovators noted the potential usefulness of HCD and systems thinking but felt that they were too far along in program development to fully utilize the methodologies. For this reason, the first iteration of design funded Innovators (referred to as cohort 3) was funded concurrent to cohort 2 of the traditionally-funded Innovators. The purpose of this new iteration was to give organizations an opportunity to develop a new idea through an intensive HCD training process rather than beginning with an already defined idea. This gave the Innovators the opportunity to fully utilize HCD throughout the entire program development process. The use of HCD earlier in the process ensured incorporation of multiple levels of feedback including target population, organizational members and leaders, community members, and others who were relevant to the process. This design process was further iterated in subsequent years.

### Design funded innovators

Cohorts 3 through 5 (2017–2020), consisted of three separate models for facilitating innovation through intensive HCD training. Cohort 3 was considered a pilot year for the design funding model, as it overlapped with the traditional model. The design funding model provided intensive CBA in HCD, in an effort to facilitate the process of innovation among program developers.

#### Cohort 3: design bootcamp cohort

Cohort 3, a Design Bootcamp, was the first iteration of the design funding model. The Design Bootcamp was created to both teach Innovators the principals of HCD and facilitate HCD over a four-day period, resulting in an innovative program. In total, two Innovators were funded as a result of one Design Bootcamp in which four organizations participated in a four consecutive day Bootcamp. iTP_3_ personnel facilitated the intensive HCD process to develop innovative ideas beginning with completing ethnography to gain insights about the target population before embarking on a process of multiples rounds of diverging and converging ideas through guided design activities to ultimately converge on one idea for each organization. Each of the four organizations pitched their program idea at the end of the Design Bootcamp, in a competition for program funding. Pitches were judged by iTP_3_ personnel, as well as community stakeholders who had knowledge of community needs and resources. The winning Innovators were given a small amount of funding to develop their program idea over a 6 month time period. They were required to have monthly check ins with an iTP_3_ liaison and report on program development and/or implementation process.

The two programs funded through the Design Bootcamp both targeted rural populations in the same community through community-based (out of school) interventions. However, the interventions were quite different. One intervention entailed adapting an existing HIV/AIDS curriculum for pregnancy prevention, using a community health worker model. The other focused on the creation of a sexual and reproductive health (SRH) game for middle school students. See Table 4 for an overview of Cohort 3 programs.

**Table 4:** Overview of Cohort 3 Innovators

Innovator	Priority population	Setting	Innovation/progress notes
**1**	Rural populations	Out of school time; Community-based	Gathered community input on development of an interactive game for middle school students; plan to continue development of a SRH game with iTP3 team
**2**	Rural populations	Out of school time; Community-based	Adapted TORO HIV/AIDS curriculum for pregnancy prevention; Began implementation of TORO curriculum locally through CHWs

### Program model iteration two

Throughout the process, it was quickly evident that requiring a full week for design work was an unrealistic expectation for many professionals. For this reason, the iTP_3_ team developed four- and two-day HCD workshops to accommodate different schedules through a Design Bootcamp model. To further address this challenge, iTP_3_ transitioned to a Design Sprint model in which full-day design sessions were separated by several weeks. The time between sessions was intended to allow for organizations to further develop their idea and gather feedback between sessions.

#### Cohort 4: design sprint cohort

Cohort 4, the Design Sprint model, utilized much of the same HCD methodology as the Design Bootcamp (i.e., ethnography followed by activities intended to cycle through rounds of diverging and converging ideas) but was divided into multiple one-day workshops over the course of several months. Organizations were supported to bring together teams of people within their local communities to participate in the Design Sprints, facilitated by iTP_3_ personnel. Therefore each of the Design Sprint funded organizations convened their own Design Sprint focused solely on the local community, in which multiple stakeholder organizations collaborated to develop a single, community-specific innovative idea. The agenda for each Design Sprint differed based on the progress and needs of the collaborative group as HCD includes an array of activities designed to facilitate innovative development. This differed from the Design Bootcamp in that organizations within a community did not compete with one another, but rather collaborated with one another.

Four distinct programs resulted from the Design Sprints in four very different communities. The resulting programs focused on training the staff of youth serving organizations, a music-based program for young males in juvenile justice settings, adult caregivers and adolescents through SRH videos, and creation of a guide for policy development for communities and schools. See Table 5 for an overview of Cohort 4 programs.

**Table 5:** Overview of Cohort 4 Innovators

Innovator	Priority population	Setting	Innovation/progress notes
**1**	Youth serving organizations	Community- based	Developed a diverse coalition of youth serving organizations, first responders, healthcare and others to connect youth to programs and providers; Focus on training of program staff
**2**	Young males; Systems-involved youth	Juvenile Justice Settings	Developed trauma informed TPP program to combine music-based activities with comprehensive SRH
**3**	Adult caregivers; adolescents	Tech	Began development of videos to prepare parents or caregivers to talk with youth about SRH topics
**4**	Organizations- community and schools	Community	Created a reference guide to help communities and schools address adolescent health and implement appropriate policies

#### Interviews

Interviews were conducted with representatives from all four Cohort 4 organizations. Cohort 4 Innovators felt that HCD and systems thinking were useful in their program development and appreciated the opportunity to bring together a diverse group with varying perspectives. Because of the fluidity and intentional flexibility of this model, each Design Sprint team participated in different activities and a process tailored to their community. For this reason, it was more difficult to identify distinct themes across Innovators. However, these Innovators recognized the need to involve stakeholders and gain priority population input, and felt that the skills gained through the Design Sprint process were not only valuable in this project, but also transferrable to other projects and could be used throughout their broader organizations.

#### After action reports

After action reports, completed by the iTP_3_ team, documented design activities completed and notes on the design session including process, engagement, reflections, pivot points, and lessons learned. In reviewing the reports, the iTP_3_ team recognized a challenge in traveling to communities to conduct Design Sprint sessions, and the desire for community members to be equipped to lead design activities and use HCD strategies in other aspects of their work. The team decided it would be more feasible to train community members to be design leaders that conduct Design Sprints with stakeholders in their community, rather than iTP_3_ traveling to communities and leading design sessions. This would increase the number of communities served and sustain the use of HCD beyond iTP_3_-funded projects.

### Program model iteration three

As iTP_3_ approached its final year, the team added a focus on sustainability of not just programs, but also equipping individuals at the local level to use HCD and systems thinking methodologies beyond this project. For this reason, Cohort 5 shifted to a Design Cadre model in which individuals convened for three in person trainings in which Design Cadre participants were trained to lead design sessions, similar to the Design Sprint process, within their own organizations or communities. Additionally, this model of training facilitators lessened the travel burden on iTP_3_ team members as the iTP_3_ team did not have to travel repeatedly to all sites to facilitate design sessions.

#### Cohort 5: design cadre cohort

The final iteration of Design Funded Innovators made the transition to empowering individuals to lead the design process within their own communities rather than relying on the external facilitation of the iTP_3_ team. This served to increase program design capacity and sustainability within communities. Eleven individuals were funded to participate in the Design Cadre to receive in person and virtual training. Following the training, they were expected to facilitate a Design Sprint consisting of a minimum total of 20 h of design work to develop a program idea within their organization or community. In total, ten organizations were represented in the design process as two individuals represented one organization. Following Design Sprint sessions conducted in communities, Cadre participants were asked to present or ‘pitch’ their initial program idea to the iTP_3_ project team. After the initial presentations, ten individuals (nine organizations) were invited to further iterate their idea and prepare a full pitch to be presented at one final in-person meeting, for a chance at receiving funding to pursue implementation of their program idea. Based on the final pitches, eight organizations were provided funding in varying amounts to continue program development activities.

Of the nine programs that were pitched, the programs focused on nine separate priority populations. Examples of priority populations identified for these programs include homeless and runaway male youth, School Based Health Center Youth Action Councils, health educators, and youth of varying age ranges. Settings represented rural and urban environments, community-based settings, school-based settings, and virtual settings. The program ideas were formulated and proposed, but not fully developed and executed due to the format of the cohort, timeline barriers imposed by the onset of COVID-19, and community-based restrictions enforced soon after the programs were funded for development. See Table 6 for an overview of Cohort 5 Innovators.

**Table 6:** Overview of Cohort 5 Innovators

Innovator	Priority population	Setting	Innovation/progress notes
**1**	Youth age 10–18	Community-based	Interactive, social exchange where young folks are able to practice asking and answering questions around sexual health, pregnancy, pregnancy prevention, STIs, access to reproductive services, etc.
**2**	Rural School District administration and/or staff; Rural Youth Program administration and/or staff	Rural Community Schools; Youth-Based Programs	Intentional approach to health education with community informed policy and implementation guidance; Guidebook helps assess both the internal and external community climate. It offers tools and strategies for developing policy with community representation.
**3**	Homeless and runaway youth, males	Outdoors, out of school/ community-based programs, runaway and homeless youth settings	Program designed for homeless and at-risk youth of all genders with a focus on parenting and care-giving youth; program emphasizes social connectedness and creates opportunities for youth to build healthy support networks with peers, social service staff, and medical providers.
**4**	Parents & Guardians	Community-based	Community health worker-led program to increase confidence and teach parents/guardians to communicate about SRH includes online toolkit and support. *Not awarded additional funds after final pitch*
**5**	SBHC YAC Coordinators, YACs, and SBHC users	School-based health centers	A collaborative youth-adult coaching model to create more powerful and effective Youth Action Councils (YACs) at school-based health centers (SBHCs).
**6**	Girls ages 13–19	Urban; school and community-based	Provide innovative health education experience through a bus equipped with latest technology to deliver age appropriate, gender-responsive, and medically accurate comprehensive sex education
**7**	Health educators	School and community- based	Equip a broader network of health educators to implement a curriculum
**8**	Youth in grades 9–11	In-school, after school, community-based	Theatre based program focused on holistic behavioral health including reproductive health, substance use prevention, and arts education
**9**	Young Adult Leaders, 13–17	Out of school time; remote, optional in-person engagement	Leadership academy to equip young people to become more confident in their ability to participate in and facilitate dialogue on SRH; program pillars focus on dialogue, values, social justice, and storytelling

#### Interviews

Interviews were conducted with 10 of the 11 Cadre participants in Cohort 5. Interviews focused on the participant’s perceptions of the design process, the resulting program, innovation, and other relevant domains. After the Cadre experience, most felt prepared to lead design sessions to facilitate innovative program development in their communities with half of participants making recommendations for additional training needs to help with full program development. All participants considered the in-person group trainings to be the most beneficial part of the experience. Within the in-person group trainings, participants identified experiencing the design methods, practicing facilitation, and one-on-one coaching as the most valuable aspects.

Cadre participants were asked to share the most important characteristics for innovation. The most common response was the need to have appropriate people involved in the design process including relevant stakeholders and end users, and ensuring that those involved are committed to the process. Participants shared that having the appropriate time to dedicate to the process and capacity to conduct the sessions was very important from a logistical perspective. Support for the process was also highlighted by participants particularly noting the importance of having buy-in from leadership, teams and the overall organization. Additionally, broad knowledge of design skills for both the facilitator and team was noted as important, as well as other individual characteristics including being willing to try something new and fail, having flexibility to change course, and being able to step back and listen.

## Conclusion

Throughout the five-year project period for iTP_3_, formal and informal data were used to refine and iterate the program model using CQI, with the goal of facilitating the development of innovative TPP programs. The resulting iTP_3_ evaluation provided a comparison between a traditional model of funding innovative ideas versus investing in building the capacity of local individuals and organizations to facilitate the development of innovative ideas.

In looking at the overall portfolio of innovative programs that was developed through iTP_3_, a total of 27 organizations were funded, with 29 programs emerging and reaching varying levels of program development. Twelve organizations received funding for multiple years, ranging from two to 4 years. Programs with potential to impact the field of TPP and adolescent sexual and reproductive health emerged from both funding models. However, equipping local individuals with skills to facilitate innovative program development was the model that ultimately resulted from years of iteration. iTP_3_ recognized this funding as a unique opportunity to fund new types of program development whereas other funding streams were already supportive of more traditional program development methods that started with a defined idea and progressed on a somewhat linear path through development and testing. While iTP_3_ was able to see the initial stages of both funding models, the timeline of the grant did not allow for many fully developed ideas that could be rigorously tested. Additionally, due to the nature of innovation, many of the resulting programs are not structured in a way to fit typical methods for rigorous evaluation. For this reason, we are not able to definitively say which model was more successful if success is defined as programs ready for rigorous testing. In order to fully test this new cohort of programs, there will need to be changes to move away from the traditional individually-focused expectations for evaluation. In total, three programs planned under the traditional funding model either received funding or applied for rigorous testing. However, none of the innovation focused funding model programs were prepared for this step. Many of these programs continued to be developed, still contributing to the portfolio of innovative programs. For this reason, it was important to look at other factors in determining potential success and impact of programs.

Goodman and colleagues identified ten domains of community capacity that can be applied in this model of equipping individuals to be better able to facilitate within their communities [19]. Through training local individuals, the iTP_3_ program addressed the dimensions of 1) participation and leadership, 2) skills, and 3) resources. With this, the local facilitator collaborates with a partner organization or network of partners engrained in the community to develop sustainable, relevant programs, both now and in the future through maximizing these and other dimensions of community capacity.

As a team, iTP_3_ decided to continue funding for promising interventions from the traditionally funded model rather than abandoning the interventions when the direction of the overall program shifted to a design-focused model. These organizations were already equipped to carry out programs, but benefited from having iTP_3_ funding as an opportunity to deviate from traditional planning methods, typically employed within their organizations.

As iTP_3_ progressed and new program development iterations were utilized, the type of programs developed changed. In the beginning, programs were highly structured, congruent with the higher funding level and traditional style of application. Some of the examples of innovative programs that emerged from the traditional funding model are as follows. Through 3 years of funding, iTP_3_ funded a(n):

1. Multi-level program to engage homeless and runaway youth with a comprehensive program that includes sexual and reproductive health (SRH), including access to long-acting reversible contraceptives (LARC) in addition to wrap around services.
2. Program working to provide capacity building through a multi-level approach to training foster care professionals, creating organizational policies and practices that support SRH education and referrals, and enhancing the physical environment of foster care agencies with SRH materials and messages.
3. Intervention for depressed young women utilizing a texting modality to provide real-time support and access to information when the information was most needed. Following iTP3, the organization received funding for rigorous testing.

In addition to the programs described above, a different set of programs emerged from the design-focused cohorts with a few notable differences. These programs were awarded less funding and the funding that was distributed was based on the design process. A few examples of the programs that emerged are described below.

1. In Cohort 3, the Design Bootcamp cohort, one organization developed a SRH game for middle school students. After several iterations of the prototype, the resulting game encompassed four games focused on knowledge and skill development regarding anatomy, healthy relationships, accessing information, and building connections with trusted adults to be equipped for navigating future relationships. This intervention was fully prototyped, pilot tested with several new communities, and ready for rigorous testing and scaling.
2. Through Cohort 4, the Design Sprint cohort, one team developed a program idea to formulate a policy guide for communities and schools. The goal of this guide was to develop a tool that organizations can use to navigate the complexities of policy development and equip practitioners with best practices and tips for working through the process.
3. In Cohort 5, the Design Cadre cohort, one organization developed an innovative health education experience utilizing a youth-friendly mobile unit to provide SRH information in a comfortable, easily accessible way. This mobile unit can travel to schools, community locations, or wherever there is a need. This program was funded for the initial development, but with the onset of COVID-19 was not fully built out prior to the end of funding.

While both types of funding models resulted in the development of innovative programs, there were notable differences. First, based on the amount of funding and the guarantee (or lack thereof) of future funding, the programs differed in scale. The programs described under the traditional funding model take more of a comprehensive approach and require much more personnel time and other resources to manage. Conversely, the design funding model resulted in programs that could be implemented and built out in phases based on funding availability. Additionally, the design-focused funding model allowed for smaller scale testing and “failure” of program components, as is typical of a design thinking process. This ensured that large amounts of time and money were not wasted on programs that were not innovative or effective for the target population.

Overall, the data indicates that innovative programs were developed as a result of iTP_3_ funding and technical assistance through both the traditional funding model and the design funding model. However, it is unclear whether the programs developed will be successful in reducing teen pregnancy or improving adolescent health as the programs are currently in the process of pursuing opportunities for rigorous testing. Several programs funded under the traditional funding model are ready for rigorous testing and have the appropriate personnel and preliminary data to do so. While the design funding model resulted in the development of innovative programs, these programs may not fit the criteria for rigorous testing in the traditional manner.

### Implications for behavioral health

This study gleans implications for funders, researchers, and practitioners in behavioral health. First, funders can utilize the information gleaned from this study to better tailor their resources to support, advocate for, and invest in innovative behavioral health projects utilizing efficient development processes, such as HCD [12, 20]. While there is a growing recognition of the need for innovation in program development, this information can specifically make the case that allowing for flexibility of program models is helpful in fostering innovation.

Next, researchers should partner with groups developing innovative programs to study and compare the process and strategies used in the development process. Such understandings could inform future program development efforts to be more efficient and cost-effective, while maintaining forward momentum. Additionally, researchers should be at the forefront of innovation funding so that research and evaluation methods can be adapted to accurately measure innovative programs. In this case, the program model and results presented here show basic adaptations in assessment which can be expanded upon in future research to ensure that innovative programs are measured according to their program characteristics rather than adhering solely to the individual-based outcomes that are traditionally used to measure TPP EBPs.

Lastly, practitioners interested in developing innovative programs can apply the lessons learned to their own work and embrace a HCD approach to develop more meaningful programs and services with their end-users. The use of HCD in this manner shows the potential to adapt business methodologies or other practices used in other fields for relevance and advancement in health. While this paper specifically makes the case for use of HCD and to a smaller extent systems thinking, openness to non-traditional methodologies can improve the interdisciplinary nature and effectiveness of health programs.

### Unexpected events impacting the process

During iTP_3_, two major unforeseen and unavoidable events limited or changed the direction of progress and should be noted.

#### Federal funding cuts

At the start of year three, iTP_3_ and all other OPA grantees in this Tier of funding received notice that funding was cut and the five-year project would end at the end of year three (June, 2018). OPA grantees did not receive notice the funding had been reinstated until June 2018; therefore, the iTP_3_ team was unable to plan for and begin year four recruitment and selection until the project year was already underway.

#### Onset of COVID-19

During year five, the COVID-19 pandemic halted the ability to complete a final systems thinking training with Design Cadre Innovators and also limited the ability of Cadre Innovators to start or continue the development of their proposed innovative program. For this reason, the iTP_3_ team was not able to fully see how the programs would be carried out, as intended. Additionally, follow-up with past Innovators was limited.

## Data Availability

The datasets analyzed during the current study are available from the corresponding author on reasonable request.

## Abbreviations

EBP: Evidence-based program
iTP3: Innovative Teen Pregnancy Prevention Programs
TPP: Teen pregnancy prevention
OPA: Office of Population Affairs
CBA: Capacity building assistance
HCD: Human-centered design
CQI: Continuous quality improvement
LGBTQ+: Lesbian, gay, bisexual, transgender plus
SRH: Sexual and reproductive health
LARC: Long-acting reversible contraceptives
